# Clinical study on horizontal bone augmentation using an alveolar mucosa-periosteal bone flap

**DOI:** 10.1186/s12903-025-05539-9

**Published:** 2025-02-08

**Authors:** Xinghuanyu Xu, Pu Xu, Shuangxi Liu, Yanlan Yang, Yanan Cheng, WenBai Zhang, Dou Yu, Liying Lu

**Affiliations:** Department of Oral Implant, Stomatology Center, Affiliated Haikou Hospital of Central South University Xiangya Medical School & Hainan Provincial Stomatology Center, No. 43 Renmin Road, Haidian Island, Meilan District, Haikou, 570208 Hainan China

**Keywords:** Alveolar ridge, Horizontal bone augmentation, Mucosa-periosteal bone flap, Dental implant

## Abstract

The current study aimed to assess the effectiveness of the alveolar bone mucosa- periosteal bone flap technique in horizontally augmenting the alveolar ridge during dental implant placement. This retrospective analysis included 20 patients with a total of 45 implants, and was designed to evaluate the alveolar ridge widths both before and after surgery. Preoperative measurements indicated an average alveolar ridge width of 3.62 ± 0.90 mm, which increased to 6.58 ± 1.16 mm postoperatively. Statistical analysis revealed a significant increase in alveolar ridge width following the procedure (*P* < 0.05), with an average gain of 2.96 ± 1.21 mm. In summary, these findings suggest that the alveolar bone mucosa-periosteal bone flap technique is an effective approach for widening the alveolar ridge while placing dental implants, meriting its consideration for clinical application.

Dental implant is a common method used to restore maxillary and mandibular missing teeth [1, 2]. To obtain the ideal osseointegration and restoration, it is required to maintain the buccal and palatal plate width of 1 mm at least [3]. Trauma, atrophy, or surgeries may lead to alveolar bone loss in certain instances, where bone augmentation has been proven effectivee [4–6].

Mucosa-periosteal bone flap (MBF) is constituted by labial (buccal) mucoperiosteal flap and bone for the sake of increasing the horizontal alveolar ridge width, which cuts a trapezoid bone block penetrating the cortex from the alveolar bone along the alveolar crest and lip (buccal) bone in the alveolar bone width deficiency region, and bone splitting is performed along the alveolar crest of primary bone cutting line after 4 weeks [7]. The purpose of this study was to evaluate the impact of alveolar ridge bone augmentation employing the MBF technique on 20 patients (45 implants), by quantifying the widths of the alveolar ridges both prior to and following implantation.

## Materials and methods

### Participants

The patients with narrow alveolar ridges(Alveolar ridge width < 2 mm) requiring dental implantation were recruited from *Oral Implantation Department, Affiliated Haikou Hospital of Central South University Xiangya Medical School & Hainan Provincial Stomatology Center* from October 2013 to December 2023. Inclusion criteria: (1) Patients who received MBF surgery to operate bone increment; (2) patients without contraindication in partial or the whole body; (3) patients not taking medicines that affected bone growth within the last 12 months; and (4) patients with complete case information without loss of follow-up.

## Instruments and materials

1. Instruments: CBCT (New Tom, Italy), piezosurgery (SATELEC Piezotome 2, France), dental implanter (Nobel, Austria) and centrifuge (TD4Z-WS, China), etc.
2. Medical appliances: mainly including Osseoset 200(Nobel, Switzerland), Nobel Active AB(Nobel, Sweden), Bicon toolbox(Bicon, USA) and other relevant appliances.
3. Implants: Nobel Active (Nobel, Sweden), Bicon (Bicon, America), Osstem (Osstem, Korea) and Dentis (Dentis, Korea).

### Treatment process

1. Periodontal non-surgical treatment: supragingival scaling, subgingival scaling and oral hygiene education were routinely carried out.
2. The PRF preparation was accomplished according to the approach introduced by *Cheng Yanan*, et al. [8].
3. The MBF technique was prepared and the implant was inserted using the method presented by *Xu Pu*, et al. [7].
4. Stage II operation and missing tooth restoration: at 3–6 months after the insertion of implants, stage II operation was performed. To be specific, the healing cap was removed, and then the healing abutments were inserted for accelerating soft tissue sulci formation. After 2 weeks, the cuffs were in healthy condition, and the missing teeth were restored after taking an impression.

### Effect evaluation

1. Clinical effect: Whether soft tissue of the cuff lip was full, and whether the fullness on the labial (buccal) side of the edentulous area was restored after the completion of stage II operation and prostheses.
2. Cone beam computed tomography (CT) observation: Cross-section changes of alveolar ridge, alveolar crest width and bone plate thickness on the lip (cheek) side after implantation.
3. Measurement of the alveolar ridge widths before and after implantation: The alveolar ridge widths were determined before implantation and after the final restoration using MBF technique. Then, data were compared.

### Statistical analysis

The SPSS Statistics software (version 17.0) was utilized for data analysis. Furthermore, paired t-tests were conducted to compare the widths of the alveolar ridges before implantation with those following the final restoration, with the outcomes presented as Mean ± Standard Deviation (M ± SD). The threshold for statistical significance was established at *P* < 0.05.

## Brief introduction of surgical procedure (Fig. 1)

**Fig. 1:**
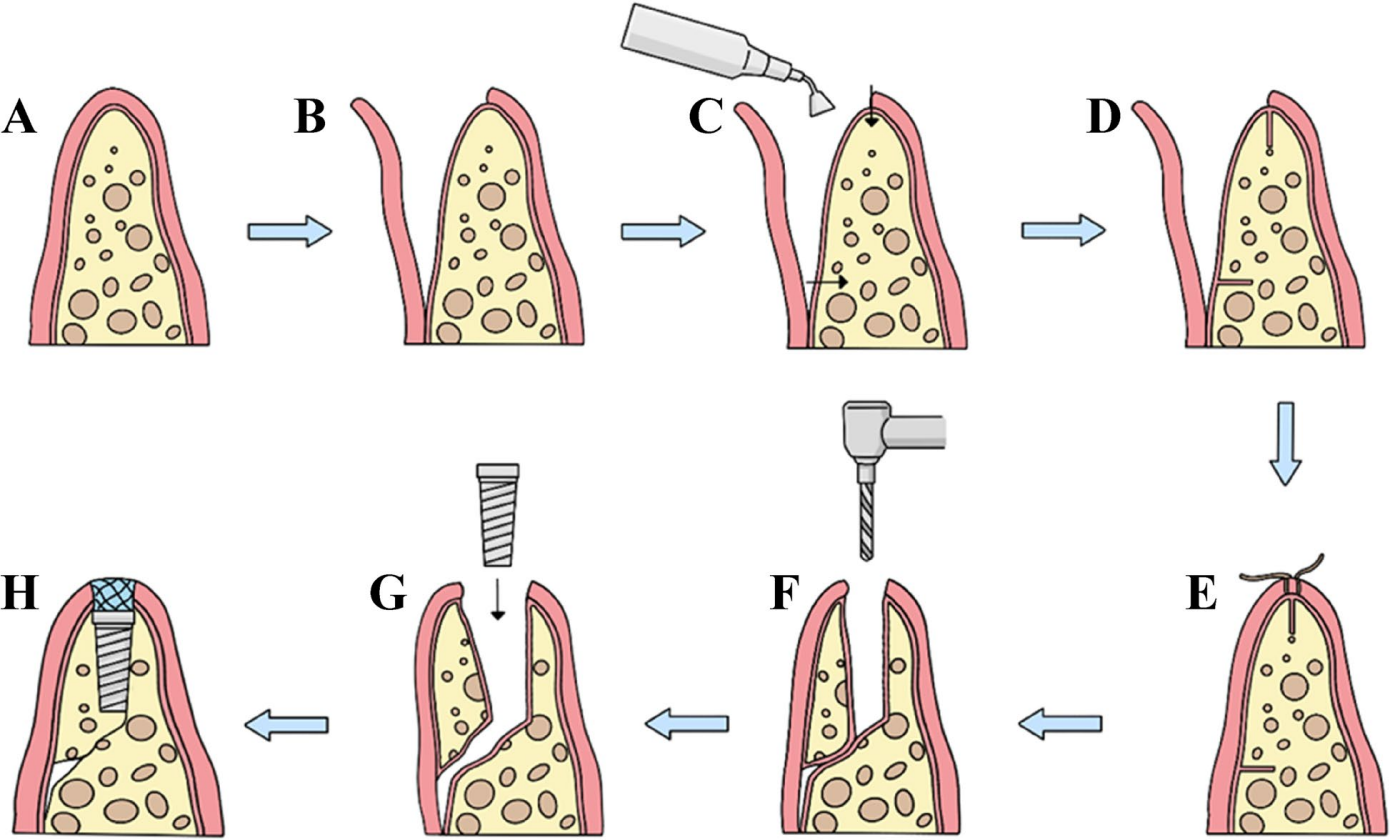
Surgical flow chart **A** Narrow alveolar ridge; **B** Turn up the mucosa-periosteal flap; **C** Osteolysis with ultrasound knife; **D** Osteolysis; **E** Soft tissue healing; **F** Mucosa-periosteal flap displacement; **G** Implant implantation; H Biofilm covering surgical area

During the initial surgery on a narrow alveolar ridge, the surgeon incises through the gum tissue to reach the periosteum, carefully elevates the fibrous bone membrane, and employs a sonic bone cutter to fashion the bone socket along the ridge's crest and sidewalls. This process involves cutting through the cortical bone to access the cancellous bone. Following the bone preparation, the surgical site is meticulously sutured. Approximately 30 days later, the dental implant I-stage surgery is conducted. This procedure involves making an incision through the soft tissue only, avoiding the need for a flap operation. After the incision, the fibrous bone membrane is gently manipulated to increase the ridge crest's width by pushing it towards the labial and buccal sides. The implant is then inserted using standard techniques, and a biomembrane is positioned at the ridge's crest to finalize the closure. Subsequent treatment protocols remain consistent with those of traditional dental implant surgery.

## Results

The present work recruited 20 cases (age, 17–69 years) and 45 implants. The proportions of female patients (13 patients, 28 implants) and patients with mandibular lesions (12 patients, 28 implants) were higher, while those of male patients (7 patients, 17 implants) and patients with maxillary lesions (8 patients, 17 implants) were lower (Table 1).

**Table 1:** The distributions of sex and tooth position in 20 patients with insufficient width of alveolar ridge cases (implants)

	group	maxilla	Mandible	total
	male	10(22)	12(22)	7 (17)
	female	10(21)	17(34)	13(28)
	total	20(43)	29(56)	49(99)

**Fig. 2:**
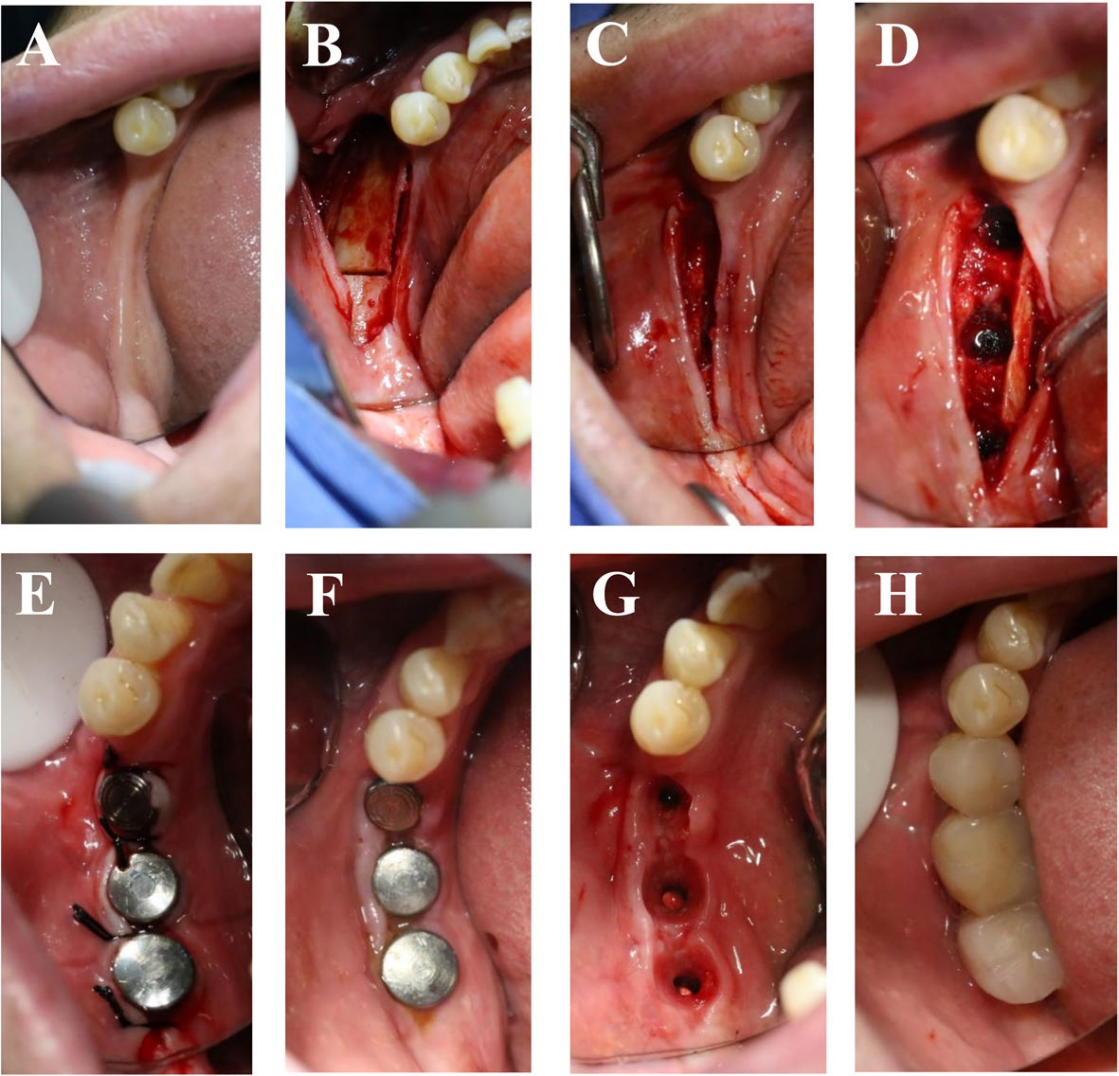
Changes in alveolar ridge before and after implantation. **A** Alveolar ridge before operation; **B** The bone is split; **C** Implant stage I surgery will be performed one month later; **D** Implant cuff; **E** Implantation phase II surgery completed; **F** Stitches removed after stage II surgery; **G** Cuff shape when the model is made; **H** Post-restoration

In clinical observation, the alveolar ridge was mostly triangular and its top was narrow before implantation. There were more soft tissues on cuff labial (buccal) side, thereby restoring the fullness of the edentulous area on the labial (buccal) side, and the aesthetic was available after the completion of stage II operation (Fig. 2).

Cone beam CT examination revealed that the triangular alveolar ridge before operation turned into the trapezoidal alveolar ridge in coronal section after operation, moreover, the bone on the lip (buccal) side of the implant was significantly greater than 1 mm (Fig. 2).

**Fig. 3:**
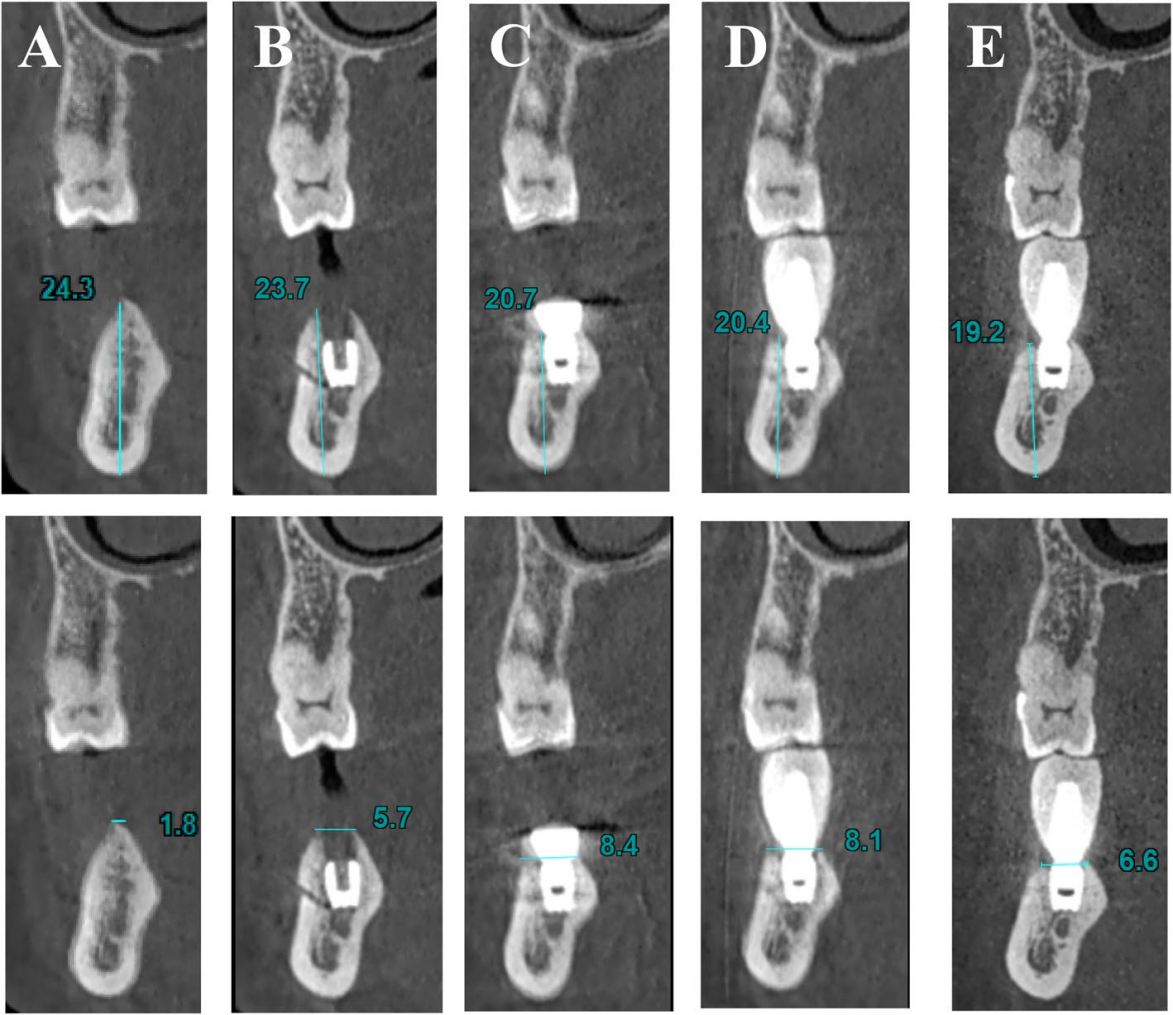
CBCT (FOV:21*19 cm, voxel: 0.3 × 0.3 × 0.3 mm) shows changes in alveolar ridge height and width. **A** Before surgery, **B** after Stage I surgery; **C** after Stage II surgery; **D** after denture; **E** Post-restoration 9 years later

Meanwhile, the alveolar ridge crest widths were determined before implantation and after the final restoration (Fig. 3), and statistical analysis was carried out. According to the results, the alveolar ridge widths were 3.62 ± 0.90 mm before implantation and 6.58 ± 1.16 mm at 3–6 months after implantation. Clearly, the alveolar ridge width elevated by an average of 2.96 ± 1.21 mm. The difference was statistically significant before and after implantation (t = 16.41, *P* = 1.573E-43 < 0.05) (Table 2).

**Table 2:** The alveolar ridge crest widths before and after implantation

	Implant number	Alveolar ridge width (M ± SD)	*P*
Before implantation	99	4.77 ± 1.49	
After restoration	99	7.68 ± 1.60	1.573E-43

## Discussion

Implant-supported restorations represent an optimal solution for replacing missing teeth, with the quality and quantity of the alveolar bone being crucial for achieving the correct implant placement [9]. Several factors may lead to inadequate alveolar ridge width, a condition that predominantly affects female patients and the mandible, as opposed to male patients and the maxilla (refer to Table 1). Asian females generally have a smaller stature compared to their European counterparts, and their skeletal structures, including the mandible, are also relatively diminutive. Patients with a narrow mandibular ridge are at a higher risk of experiencing insufficient alveolar ridge width post-tooth extraction. Given that the supporting area is smaller than that of the maxilla, the mandibular alveolar ridge is more susceptible to constriction and resorption following restoration.

The traditional osteotomy technique has proven effective in expanding alveolar bone width; however, it carries inherent risks such as fracture, necrosis, and bone resorption. In contrast, the application of a Mucosa-periosteal bone flap (MBF) yields superior results for horizontal alveolar bone augmentation. By positioning the pre-shaped trapezoidal bone block on the buccal aspect, the vascular connection between the mucoperiosteum and the bone block is preserved.This phenomenon ensures the nutritional supply to the bone graft, thereby preventing its necrosis and resorption [7].

Tooth loss often results in the diminishment of alveolar bone, leading to significant atrophy and the formation of a blade-shaped alveolar ridge [8, 10, 11]. Repairing a missing posterior tooth in an atrophic alveolar ridge presents a common clinical challenge [12–14]. During the clinical observation of this study, it was noted that the alveolar ridge predominantly exhibited a triangular shape with a narrow apex prior to implantation. The application of the MBF technique at the time of implant placement resulted in an increase in the width of the alveolar ridge's apex. After the stage II procedure, the soft tissues on the labial (buccal) side of the cuff were observed to be fully restored, effectively enhancing the fullness on the labial (buccal) aspect of the missing teeth. As a result, the aesthetic outcome was considered satisfactory following the teeth restoration (Fig. 1).

The cone beam CT examination demonstrated that the implant restoration cases in this study resulted in favorable clinical outcomes. Post-implantation, the alveolar ridge was notably broadened, exhibiting a trapezoidal cross-section, in contrast to its pre-implantation triangular cross-section. Additionally, the bone on the buccal side of the implant was observed to be significantly more than 1 mm. Upon measurement, the alveolar ridge widths were found to be 3.62 ± 0.90 mm prior to implantation and 6.58 ± 1.16 mm following implantation, indicating an increase of 2.96 ± 1.21 mm. The alveolar crest width experienced a significant augmentation post-operation, as depicted in Fig. 2. These findings suggest that the MBF technique yields positive clinical effects, aligning with the outcomes reported by other researchers [15].

Some researchers have indicated that bone grafting can achieve superior outcomes in the enhancement of alveolar bone [16, 17]. However, it is crucial to create secondary surgical sites to obtain graft material. Implant placement is postponed for 3–6 months, and the graft bone absorption rate reaches 20%-50% at 6 months later [18]. The guided bone regeneration (GBR) procedures carry the risk of membrane exposure and rupture, which can result in infection [15, 19]. Inter-positional augmentation, which includes ridge split and ridge expansion, has achieved optimal results in the maxilla [14]. However, in the mandible, there is an elevated risk of block fracture post-osteotomy due to the dense cortical bone. To minimize the risk of fractures in these patients, apical and vertical horizontal cuts are executed. The ridge split procedure exhibits flexibility when immature bone callus forms at the cortical incision site. Certain scholars suggest that the ridge split can be conducted once the callus has matured (typically at 3 months) at the cortical incision site, resulting in improved primary stability of the implants [20].

In general, the task of cutting cortical bone with manual instruments during the MBF operation is challenging, especially when it comes to achieving precise osteotomy. The employment of motor-driven equipment for bone cutting generates considerable heat, which can prolong healing times due to the adverse effects on surrounding tissues. Consequently, the piezoelectric osteotomy technique presented in this article offers two significant benefits. First and foremost, the precision of the cut is ensured, as the ultrasound frequency used does not harm the soft tissues. Secondly, the procedure is less invasive, leading to improved healing outcomes. This method yields a more predictable healing effect…[21].

In conclusion, the MBF technique consistently improves the width of the alveolar bone and utilizes piezoelectric osteotomy to achieve the desired outcomes. When creating the "mucous bone flap," it is crucial to consider the labial bone thickness to ensure osteogenesis in the lip bone of the implant, thereby enhancing the implant restoration outcomes both in the short and long term [7]. The MBF technique presents a novel strategy for addressing inadequate alveolar ridges. However, the study is not without its limitations, including a limited sample size, absence of a control group, and the necessity for an extended duration to monitor long-term outcomes. Moving forward, subsequent research will persist in comparing case numbers and other bone augmentation methodologies.

## Data Availability

The datasets generated and/or analyzed during the current study are not publicly available due [The project is not finished] but are available from the corresponding author on reasonable request.

## Abbreviations

MBF: Mucosa-periosteal bone flap
CT: Computerized tomography
